# Malaria in HIV-Infected Children Receiving HIV Protease-Inhibitor- Compared with Non-Nucleoside Reverse Transcriptase Inhibitor-Based Antiretroviral Therapy, IMPAACT P1068s, Substudy to P1060

**DOI:** 10.1371/journal.pone.0165140

**Published:** 2016-12-09

**Authors:** Charlotte V. Hobbs, Erin E. Gabriel, Portia Kamthunzi, Gerald Tegha, Jean Tauzie, Elizabeth Petzold, Linda Barlow-Mosha, Benjamin H. Chi, Yonghua Li, Tiina Ilmet, Brian Kirmse, Jillian Neal, Sunil Parikh, Nagamah Deygoo, Patrick Jean Philippe, Lynne Mofenson, William Prescott, Jingyang Chen, Philippa Musoke, Paul Palumbo, Patrick E. Duffy, William Borkowsky, for the P1068s Study Team

**Affiliations:** 1 Laboratory of Malaria Immunology and Vaccinology, National Institute of Allergy and Infectious Diseases, National Institutes of Health, Rockville, MD, United States of America; 2 Department of Pediatrics, Division of Infectious Disease and Immunology, New York University School of Medicine, NY, United States of America; 3 Batson Children’s Hospital, Department of Pediatrics (Division of Infectious Diseases) and Department of Microbiology, University of Mississippi Medical Center, Jackson, MS, United States of America; 4 Biostatistics Research Branch, National Institute of Allergy and Infectious Diseases, National Institutes of Health, Rockville, Maryland, United States of America; 5 Kamuzu Central Hospital, University of North Carolina at Chapel Hill Lilongwe Project, Lilongwe, Malawi; 6 Duke Clinical Research Institute, Durham, NC, United States of America; 7 Makerere University-Johns Hopkins University Research Collaboration, Kampala, Uganda; 8 Department of Obstetrics and Gynecology, University of North Carolina at Chapel Hill, Chapel Hill, NC, United States of America; 9 Cornell Clinical Trials Unit, Weill Cornell Medicine, NY, United States of America; 10 Department of Pediatrics, Division of Medical Genetics, University of Mississippi Medical Center, Batson Children’s Hospital, Jackson, MS, United States of America; 11 Yale Schools of Public Health and Medicine, New Haven, Connecticut, United States of America; 12 HJF-DAIDS, a Division of the Henry M. Jackson Foundation for the Advancement of Military Medicine, Inc., Contractor to NIAID, NIH, DHHS, Bethesda, MD, United States of America; 13 Elizabeth Glaser Pediatric AIDS Foundation, Washington, DC, United States of America; 14 HYDAS World Health, Inc., Hummelstown, PA, United States of America; 15 Ben Towne Center for Childhood Cancer Research, Seattle Children’s Research Institute, the University of Washington, and the Fred Hutchinson Cancer Research Center, Seattle WA, United States of America; 16 Department of Pediatrics and Child Health, Makerere University, Kampala, Uganda; 17 Division of Infectious Diseases and International Health, Geisel School of Medicine at Dartmouth, Lebanon, New Hampshire, United States of America; Rush University, UNITED STATES

## Abstract

**Background:**

HIV and malaria geographically overlap. HIV protease inhibitors kill *malaria* parasites *in vitro* and *in vivo*, but further evaluation in clinical studies is needed.

**Methods:**

Thirty-one children from Malawi aged 4–62 months were followed every 3 months and at intercurrent illness visits for ≤47 months (September 2009-December 2011). We compared malaria parasite carriage by blood smear microscopy (BS) and confirmed clinical malaria incidence (CCM, or positive BS with malaria symptoms) in children initiated on HIV antiretroviral therapy (ART) with zidovudine, lamivudine, and either nevirapine (NVP), a non-nucleoside reverse transcriptase inhibitor, or lopinavir-ritonavir (LPV-rtv), a protease inhibitor.

**Results:**

We found an association between increased time to recurrent positive BS, but not CCM, when anti-malarial treatment and LPV-rtv based ART were used concurrently and when accounting for a LPV-rtv and antimalarial treatment interaction (adjusted HR 0.39; 95% CI (0.17,0.89); p = 0.03).

**Conclusions:**

LPV-rtv in combination with malaria treatment was associated with lower risk of recurrent positive BS, but not CCM, in HIV-infected children. Larger, randomized studies are needed to confirm these findings which may permit ART optimization for malaria-endemic settings.

**Trial Registration:**

ClinicalTrials.gov NCT00719602

## Introduction

Malaria is highly prevalent in many areas of the world where HIV-infected children live, especially sub-Saharan Africa. Studies have shown that when HIV and malaria are present as co-infections, each disease can enhance the pathogenicity of the other[1]. Moreover, as more patients are managed for HIV infection in malaria endemic areas, understanding HIV drug impact on malaria infection is important. If selecting certain antiretroviral regimens over others may not only treat HIV but also potentially reduce malaria burden, such approaches could be employed as an additional intervention to reduce malaria burden where HIV and malaria are co-endemic.

The World Health Organization (WHO) recommends HIV management with combination antiretroviral therapy (ART), with first line therapy including a non-nucleoside reverse transcriptase inhibitor (NNRTI) and 2 nucleoside reverse transcriptase inhibitors (NRTIs), with few exceptions, and second line therapy including an HIV protease inhibitor (HIV PI) and 2 NRTIs [2]. Both *in vitro* and *in vivo* data with *Plasmodium* suggest that HIV PIs kill various life cycle stages of malaria parasites [3–7], indicating these drugs may influence malaria infection in people. Indeed, a clinical study in which children who were randomized to receive protease inhibitor lopinavir-ritonavir (LPV-rtv)-based antiretroviral therapy (ART) had fewer cases of recurrent clinical malaria when compared with NNRTI nevirapine (NVP)-based ART in an area of high malaria transmission intensity [8].

Because transmission intensity influences the dynamics of malaria infection and intervention efficacy [9], we sought to quantify the impact of different antiretroviral regimens on parasite carriage in HIV-infected children by microscopy and clinical malaria in the first such study performed in children in an area of low to moderate transmission. Herein we describe malaria infection in children who received either LPV-rtv based ART (LPV-rtv ART) or NVP-based ART (NVP ART) as treatment for HIV infection in the clinical substudy P1068s, which was a substudy to the larger, randomized HIV pediatric treatment study, P1060 [10, 11].

## Methods

### Study Population

P1068s was substudy to the International Maternal Pediatric Adolescent AIDS Clinical Trials Network (IMPAACT) study P1060, details of which have been reported elsewhere [10, 11]. Briefly, P1060 was an HIV treatment study conducted at 6 countries in sub-Saharan Africa and India that enrolled HIV-infected children aged 2 months-36 months who qualified for treatment according to World Health Organization (WHO) criteria. Children were randomized to receive zidovudine and lamivudine combined with either NVP or LPV-rtv, and were followed for < = 47 months (median follow-up 32 months), and stratified by exposure to NVP at birth and by age (<1 year vs. > = 1 year) in the context of P1068s. ART failure was defined as permanent discontinuation of the treatment regimen for any reason, including the need for treatment of tuberculosis during the course of the study [10, 11].

The malaria substudy described herein, P1068s, was conducted at three sites with endemic malaria transmission according to published data at the time, which included Kampala, Uganda; Lusaka, Zambia; and Lilongwe, Malawi [12–14]. Analysis was performed only on data from the Malawi site, however, because of low blood smear positivity rates at the other sites: among the 74 subjects enrolled at the Zambia and Uganda sites, only 8 subjects had a positive blood smear over the course of follow-up for a total of 19 positive BS over the combined 4 year accrual and follow-up period, although one child in Uganda died from unconfirmed but suspected severe malaria [11].

All participants from the parent HIV treatment study, P1060 were eligible for inclusion in this malaria substudy. Although not randomized for the primary objectives of the P1068s substudy described herein, it was originally intended the randomization of P1060 would be used for P1068s; however, due to later initiation of this substudy, a number of subjects had switched from the regimen to which they had been originally randomized. Thus, most data collected under P1068s that links treatment to outcome is observational and most analysis was conducted using an “as-treated” approach.

### Follow-Up

Study visits were conducted 2 and 4 weeks after the initiation of treatment, every 4 weeks until week 16, at week 24, and every 12 weeks thereafter, and patients were encouraged to come in for intercurrent illness. Follow up for P1068s paralleled P1060 (Table 1).

**Table 1:** Demographic Information for Children Enrolled into P1068s from Lilongwe, Malawi.

Characteristics	LPV-rtv ART only, N = 16	NVP ART only, N = 7	Switched from NVP ART to LPV-rtv ART, N = 8
Male # subjects (%)	7 (43.8%)	4 (57.1%)	3 (37.5%)
Enrollment Age (months), Median (min, max)	28.4 (3.9, 61.3)	33.0 (24.8, 51.9)	33.9 (25.8, 61.8)
Duration of Follow-Up on P1068s (months), Median (min, max)	42.7 (22.3, 46.8)	43.1 (20.7,45.4)	36.8 (20.9,46.8)
Duration on LPV-rtv ART during P1068s (months), Median (min, max)	42.7 (22.3, 46.8)	--	24.4 (8.6, 42.6)
Follow-Up Time Prior to Switching ART regimen (months), Median (min, max)	42.7 (22.3, 46.8)	43.1 (8.3, 45.4)	1.2 (0.0,14.5)
Positive Blood Smear Count, Median (min, max)	2.5 (0.0, 16.0)	3.0 (0.0, 19.0)	3.0 (0.0, 5.0)
Count Confirmed Clinical Malaria, Median (min, max)	2.5 (0.0, 15.0)	3.0 (0.0, 15.0)	2.5 (0.0, 5.0)
Months on P1060 before P1068s enrollment, Median (min, max)	12.2 (8.2, 32.6)	11.9 (9.6, 34.3)	12.2 (8.2,34.2)

History and physical exams were completed at all scheduled and intercurrent illness visits, and children were seen by study physicians at all visits. Heel or finger stick or venous blood were used to prepare thick blood smear using 2% Giemsa for 30 minutes, in addition to dried blood spots (DBS, with 50 µL/spot) collected on Whatman 903 paper (Florham Park, NJ) and handled as previously described [15] for confirmatory PCR (see Supporting Information, S1 File [16, 17]). CD4 cell count, CD4%, and HIV viral load were measured in real time during the P1060 trial [11]. Subjects were diagnosed with malaria on site and treated as per WHO recommendations, which for this study included artemether-lumefantrine for uncomplicated malaria and quinine or artesunate for severe malaria [18].

### Study Site

In Malawi, trimethoprim-sulfamethoxazole prophylaxis was administered as per WHO guidelines [19, 20], and all patients received prophylaxis. At the beginning of the study, the infant feeding policy was exclusive breastfeeding recommended for the first 6 months, which changed to the first 12 months of life as per WHO, in 2010 [21], but this was not adopted by Malawi until July 2011. All children on study were given an insecticide-treated bednet at the beginning of the study and lived within 30 km of the study site. Clinical illness was managed based on the Integrated Management of Childhood Illness Guidelines [22].

Malaria transmission in Malawi is perennial and holoendemic, with seasonal increases after the rains from November to April. Malaria mapping analysis in Malawi showed lack of significant changes in transmission between 2000–2010, with a population-adjusted *P*. *falciparum* rate (*PAPfr*_*2-10*_) in 2010 of 32%; this encompasses part of the period in which this study was conducted [23].

### Ethics

This substudy was approved by site-specific institutional review boards (IRBs), including the New York University School of Medicine (NYU) IRB (January 17, 2008) and the Malawian Ministry of Health and Population National Health Sciences Research Committee (June 8, 2009) and the NIH/NIAID IRB through a reliance agreement with NYU IRB (May 18, 2012). Each child’s parent or legal guardian provided written informed consent. The study was first opened to accrual on August 19, 2009 with the first patient enrolled September 25, 2009.

### Laboratory Procedures

A blood smear was deemed negative if no parasites were seen in > = 200 high-powered fields. All blood smears in the study, with the exception of one *P*. *malariae* sample from Lilongwe, were reported as *P*. *falciparum*. The diagnosis and management of malaria was based readings of blood smears on site, but reported results herein are from reads performed at NYU, with a primary and secondary reads by two microscopists, with discrepant results resolved and a random subset of smears reviewed by a third microscopist.

Quality control of blood smear reading was maintained by 30 hours of training which covered both slide preparation, cover slipping, microscope care and maintenance and identification and quantification of malaria parasites, both at NYU and at study sites. Over the time period of the study the NYU microscopists were proficiency tested four times using archived smears provided by Hydas World Health (http://hydasworldhealth.org/), whose stock smears are read by WHO Level 1-Certified microscopists [24]. Each examination consisted of 15 cover-slipped, Giemsa stained blood smears containing thick and thin blood smears from malaria infected and uninfected blood. Blood smears included either single or mixed infections of *Plasmodium falciparum*, *vivax*, *ovale or malariae*. For the total 60 test slides, the readers’ overall sensitivity was 100%, specificity 92%, and density determination was within +/-25% of the accepted value in 96% of the slides. During the final 2 years of the study, the sensitivity and specificity were both tested at 100%. Separately, DNA was isolated from DBS and parasite genomic DNA was detected using real time from DBS collected for confirmation in parallel to smears.

### Statistical Methods

The primary end points were the time to first or recurrent events of and the rate of positive malaria blood smear (BS) or confirmed clinical malaria (CCM), defined as a positive BS with diagnosed malaria symptoms, as per WHO guidelines [18]. Only episodes of CCM greater than 14 days apart were considered, as those were assumed to represent new infections rather than recrudescence [9].

Thirty-one of 288 subjects from the P1060 parent study enrolled in P1068s, and 8 of these 31 children changed regimens based on the primary study before or after enrollment. For this reason we undertook an as-treated analysis, attempting to link observed treatment and observed outcomes. Per-protocol analysis, censoring subjects when they switched regimens, and as-randomized analysis (as randomized for the P1060 parent study) analysis are also provided in Supporting Information (S2 File and S1 and S2 Tables).

During this study, another clinical trial suggested that a drug interaction between LPV-rtv and lumefantrine (the second component of artemether-lumefantrine, used for treatment of uncomplicated malaria) reduced clinical malaria burden in children on LPV-rtv ART in an area of high transmission intensity [8]. For this reason, the relationship between time to recurrent episodes of malaria (positive BS or CCM) and ART and potential synergy between HIV ART and malaria treatment is the primary scientific question evaluated in this report. This analysis was conducted by fitting a recurrent event (count process) Cox model [25]. Malaria treatment is considered a time-varying indicator in the models, until the occurrence of a malaria event, censoring by ART change or censoring by the end of follow up. Both malaria treatment and ART regimen were treated as time-varying covariates and models were adjusted for average CD4% over prior period, sex, age at enrollment, months since start of enrollment into P1060 to start of P1068s and an indicator for malnutrition status (as defined by mid-upper arm circumference [26]) collected at each study visit.

In addition to evaluation of recurrent malaria events, comparisons of the rate of incident positive BS and CCM per time at risk by HIV treatment type was analyzed using negative binomial models including an offset for time-on-trial and adjusted for potential confounders. HIV treatment was accounted for using the following three time categories of LPV-rtv ART use: LPV-rtv ART assigned at P1060 baseline; LPV-rtv ART duration in months; or majority of time on LPV-rtv ART

Reported p-values are not corrected for the number of analyses conducted. Statistical analysis was performed with R software, version 3.1.3.

## Results

**Fig 1:**
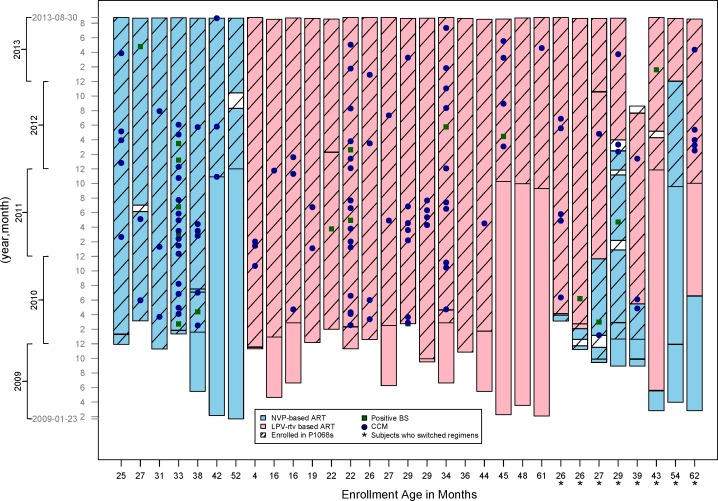
Graphic representation of patients enrolled in our substudy, P1068s, by sample date. Lopinavir-ritonavir based antiretroviral therapy (LPV-rtv ART) or NNRTI nevirapine-based ART (NVP ART) treatment regimen and outcomes of Positive Blood Smear (BS) and Confirmed Clinical Malaria (CCM) or are sorted youngest to oldest enrollment age on the x-axis. Asterisks (*) indicate subjects whose regimens went from NVP to LPV-rtv ART on study due to virologic failure as dictated by the parent study, P1060.

From Kamuzu Central Hospital, Lilongwe, Malawi, 31 children were enrolled between September 2009 and December 2011, and demographic information for these patients is summarized in Table 1. Eight patients who were randomized to start on NVP ART switched to LPV-rtv ART due to HIV treatment failure based on criteria for virologic failure as defined by the parent study P1060, with two subjects switching before enrollment into P1068s (Fig 1). One patient withdrew due to moving too far from the study site to be able to attend regular visits. There were no significant differences between the cohorts at P1060 initial randomization to LPV-rtv ART compared with NVP ART with regard to age at entry, sex or time from P1060 enrollment to P1068s enrollment, and baseline CD4% (all p-values >0.15). Adverse events were not analyzed separately for P1068s as they have been previously reported for P1060 [10, 11]. Kaplan Meier curves for time to 1^st^ positive BS and CCM are presented in Supporting Information, S1A and S1B Fig. For concurrent smear and PCR samples, the phi coefficient (or measure of association for our two binary variables) was 0.85.

In the as-treated recurrent event analysis, time on LPV-rtv ART prior to positive BS or CCM was not significantly different from NVP ART (Tables 2 and 3 Ignoring Malaria Treatment), although the Hazard Ratio (HR) of 0.71, 95%CI (0.37,1.37); p = 0.31 suggested a trend towards increased time to recurrent positive BS episodes in children taking LPV-rtv ART compared to NVP ART. Including an indicator for malaria treatment yes/no (Tables 2 and 3 Adjustment for Malaria Treatment) did not change this finding. However, when including an indicator for malaria treatment and an interaction for malaria treatment and current LPV-rtv ART (Tables 2 and 3 Malaria Treatment with Interaction), there was a significant association between malaria treatment with LPV-rtv ART use and reduced hazard of positive BS as compared to malaria treatment and current NVP ART, or even current LPV-rtv ART use without malaria treatment. The estimated HR for a positive BS for the concurrent malaria and HIV treatment group from the fully adjusted model was 0.39, 95% CI (0.17,0.89); p = 0.03. A similar interaction model for the CCM outcome did not yield significant results (HR 0.53, 95% CI (0.17,1.61); p = 0.26). The model also suggested malaria treatment was significantly associated with increased hazard of positive BS (HR 2.48, 95%CI (1.13,5.43); p = 0.02).

**Table 2:** As Treated Recurrent Events of Positive Blood Smears (BS) (Cox Model).

	Ignoring Malaria Treatment			Adjustment for Malaria Treatment			Malaria Treatment with Interaction*		
	HR	95% CI	p-value	HR	95% CI	p-value	HR	95% CI	p-value
LPV-rtv ART**	0.71	0.37,1.37	0.31	0.74	0.38,1.42	0.36	1.53	0.63,3.71	0.35
Malaria Treatment				1.31	0.84,2.06	0.23	2.48	1.13,5.43	0.02
Malnutrition (yes/no)	0.98	0.59,1.63	0.95	1.02	0.57,1.84	0.94	1.09	0.61,1.94	0.77
Mean CD4%	1.04	1,1.08	0.03	1.04	1.01,1.08	<0.001	1.04	1.01,1.08	<0.001
Enrollment Age (months)	1.26	0.87,1.84	0.22	1.28	0.9,1.83	0.17	1.23	0.87,1.75	0.24
Months on P1060 before P1068s enrollment	0.95	0.91,1	0.06	0.95	0.91,1	0.04	0.96	0.91,1	0.05
LPV-rtv/malaria treatment (interaction)							0.39	0.17,0.89	0.03

*Interaction term taken into account in analysis for LPV-rtv and malaria treatment

**LPV-rtv ART versus NVP periods of no treatment removed

**Table 3:** As Treated Recurrent Events of Confirmed Clinical Malaria (CCM) (Cox Model).

	Ignoring Malaria Treatment			Adjustment for Malaria Treatment			Malaria Treatment with Interaction*		
	HR	95% CI	p-value	HR	95% CI	p-value	HR	95% CI	p-value
LPV-rtv ART**	0.81	0.43,1.53	0.52	0.86	0.44,1.68	0.66	1.46	0.43,5.02	0.55
Malaria Treatment				1.23	0.71,2.14	0.47	2	0.63,6.34	0.24
Malnutrition (yes/no)	1.04	0.59,1.83	0.89	1.07	0.54,2.1	0.85	1.13	0.58,2.18	0.72
Mean CD4%	1.04	1,1.07	0.03	1.05	1.01,1.08	<0.001	1.04	1.01,1.08	0.01
Enrollment Age (months)	1.26	0.85,1.85	0.25	1.3	0.89,1.91	0.18	1.28	0.87,1.88	0.21
Months on P1060 before P1068s enrollment	0.96	0.91,1.01	0.08	0.95	0.91,1	0.05	0.95	0.91,1	0.05
LPV-rtv/malaria treatment (interaction)							0.53	0.17,1.61	0.26

*Interaction term taken into account in analysis for LPV-rtv and malaria treatment

**LPV-rtv ART versus NVP periods of no treatment removed

The results for the as-treated negative binomial fits of the count of positive BS and CCM using all the data and accounting for observed LPV-rtv ART use suggested that there were no significant differences in incident positive BS and CCM. However, when including an additional covariate which described changing from NVP ART to LPV-rtv ART during P1068s, we found a borderline significant association of LPV-rtv ART compared to NVP-ART with positive BS (RR 0.49 95% CI (0.25,0.98); p-value 0.05 (Table 4). The other two approaches for accounting for observed LPV-rtv ART use, duration (months) and majority LPV-rtv ART use over follow-up did not provide evidence for an association with malaria measures BS or CCM (data not shown). None of the models for CCM rate suggested that there was evidence of a difference between those subjects that were observed to use LPV-rtv ART and those that were not (All p-values > 0.1, data shown for including LPV-rtv ART assigned at P1060 baseline).

**Table 4:** As Treated Rates of Positive Blood Smears (BS) and Confirmed Clinical Malaria (CCM) (Negative Binomial Model).

	Positive BS				CCM	
Variable	RR	95% CI	p-value	RR	95% CI	p-value
LPV-rtv ART at Baseline	0.49	0.25,0.98	0.05	0.53	0.25,1.13	0.1
Switched ART regimens while on study	0.59	0.27,1.32	0.2	0.54	0.22,1.31	0.17
CD4% at enrollment	1.05	1.02,1.08	<0.01	1.05	1.02,1.08	0
Enrollment Age (months)	1.01	0.98,1.05	0.45	1.02	0.98,1.06	0.38
Sex (female)	1.4	0.79,2.48	0.25	1.52	0.81,2.85	0.19
Enrollment time between P1060 and P1068s	0.94	0.88,0.99	0.03	0.94	0.88,1	0.05

## Discussion

In our P1068s substudy, which was the first of its kind conducted in an area of low to moderate malaria transmission intensity, LPV-rtv in combination with malaria treatment is associated with lower risk of recurrent events of BS, but not CCM, in HIV-infected children.

HIV protease inhibitors are aspartyl protease inhibitors, and have been hypothesized to exhibit the killing activity for *Plasmodium* species in the laboratory because of inhibition of these enzymes, of which there are 10 in *Plasmodium falciparum*, although further investigation is required to clarify the target [6, 27, 28]. The reduced frequency of positive BS when accounting for malaria treatment, and the trend in reduced episodes of CCM and overall trend of reduced BS and CCM, could be because of direct drug killing or pharmacokinetic interactions [8, 29], the latter especially since our strongest trends were observed in recurrent events analysis which took antimalarial treatment interaction into account. Indeed, it is possible that we saw a modification in blood smear (BS), but not confirmed clinical malaria (CCM) outcome, because there were more positive BS, increasing our power to detect a difference. It is also possible that there is a modification of immune responses to malaria, either through better control of HIV or from LPV-rtv ART-mediated killing of malaria parasites, that accounts for these findings.

A previous study that was conducted in an area of high-intensity transmission has shown similar trends in children: HIV PIs were associated with an overall reduction in malaria for children on LPV-rtv ART [8]. This finding was at least partly attributed to an interaction between the ritonavir of LPV-rtv and lumefantrine component of the artemisinin-combination regimen used to treat clinical malaria [8, 30], and other studies have shown interaction between NNRTIs and antimalarial therapy [29]. Although our study is clearly distinguished by transmission intensity, which itself affects the efficacy of interventions, given factors such as differences in acquired immunity and parasite resistance [9], both studies found reduction in malaria burden with LPV-rtv ART. This is highly significant because similar studies in adults and pregnant women have not found such trends [31–33]. The trend to reduced malaria episodes in LPV-rtv ART-treated children in our study and in the work of others across varying degrees of transmission intensity [8], compared with the lack of such findings in adults and pregnant women [31–33] may suggest there are unique reasons why children may benefit from such a regimen in a malaria-endemic area, such as differences in age-related anti-malaria immunity or differential pharmacokinetics.

Of note, the as treated recurrent events model also showed that malaria treatment was significantly associated with increased hazard of repeated positive BS and CCM in the presence of NVP ART. This finding was likely due to the strong correlation between receiving a malaria treatment in the current period and having a malaria event in the previous period. We investigated this by including malaria treatment in the model only if there was not a malaria event in the previous period for a subject. This modified version of the model suggested that malaria treatment was associated with reduced hazard of malaria outcomes under all ART regimens, albeit non-significantly due to the small number episodes observed (data not shown). Therefore, one might describe our malaria treatment measure as a mixture of previous illness and malaria treatment.

A limitation of our study is our small sample size and the number of children who switched ART regimens. However, because we detected significant findings in a small sample size, and over a long period of time, we believe the effect of LPV-rtv ART may be considerable and as such, a larger study is warranted to investigate and validate this finding.

WHO Guidelines now support the use of LPV-rtv ART in children less than 3 years of age because of longer life-expectancy and cost-effectiveness compared to NNRTI ART [2]. Whether LPV-rtv ART regimens should be recommended in all children in malaria endemic areas because of additional benefits of reduced malaria remains to be clarified [34]. Further, larger, randomized studies are needed to answer these questions, including assessment of whether LPV-rtv has direct killing effects on the parasite, how this impacts acquired malaria-specific immunity, and how drug interactions affect malaria burden in HIV-infected patients. Malaria eradication will only likely be achieved by a combination of interventions (such as insecticide treated bednets, intermittent preventive treatment, and hopefully, an effective vaccine). Optimizing HIV ART to reduce malaria burden may ultimately contribute to eradication efforts.

## Supporting Information

S1 Fig **A and B: Kaplan Meier Curves depicting time to first (1**^**st**^**) positive blood smear (BS) and time to 1**^**st**^ **confirmed clinical malaria (CCM) case.** Overall, we observe a BS positive rate of 0.55 and 0.87 for the 1st year and the 1st two years of follow-up, respectively; for CCM rates are, 0.52 and 0.77 for the 1st year and the 1st two years, respectively. We observe 123 positive BS events, and 109 CCM events over the 97.89 observed person-years. In 1A, shown is the survival proportion and times to 1^st^ positive BS. The median survival times are 408 and 135 days in the randomized to-LPV-rtv ART and randomized to-NVP ART groups, respectively. However, there was an 0.88 infection rate in the LPV-rtv ART group, and an 0.87 rate in the NVP-ART group over follow-up. In 1B, shown is the survival proportion and times to 1^st^ CCM. The median time to 1^st^ CCM, is 417 and 135 days in the randomized to-LPV-rtv ART and randomized to-NVP ART groups, respectively. However, there was an 0.81 CCM rate in the LPV-rtv group, and a 0.73 rate in the NVP-based group over follow-up. Note that for BS, the longest period while on NVP-ART prior to 1st positive BS is shorter compared with LPV-ART, and the figure incorporates subject censoring.
(TIFF)

S1 File Methods, Real Time PCR. (DOC)

S2 File Additional Statistical Analysis. (DOC)

S1 Table As-randomized in P1060 analyses (A. Cox Model and B. Negative Binomial).
(DOC)

S2 Table Per Protocol Rates of BS and CCM (Negative Binomial Model, Data Censored at Switch from Regimen As Randomized). (DOC)
