# Supplementary figures and images for: Malaria in HIV-Infected Children Receiving HIV Protease-Inhibitor- Compared with Non-Nucleoside Reverse Transcriptase Inhibitor-Based Antiretroviral Therapy, IMPAACT P1068s, Substudy to P1060
Source: PLoS One. 2016 Dec 9;11(12):e0165140. doi: 10.1371/journal.pone.0165140 (PMC5147802; doi:10.1371/journal.pone.0165140)

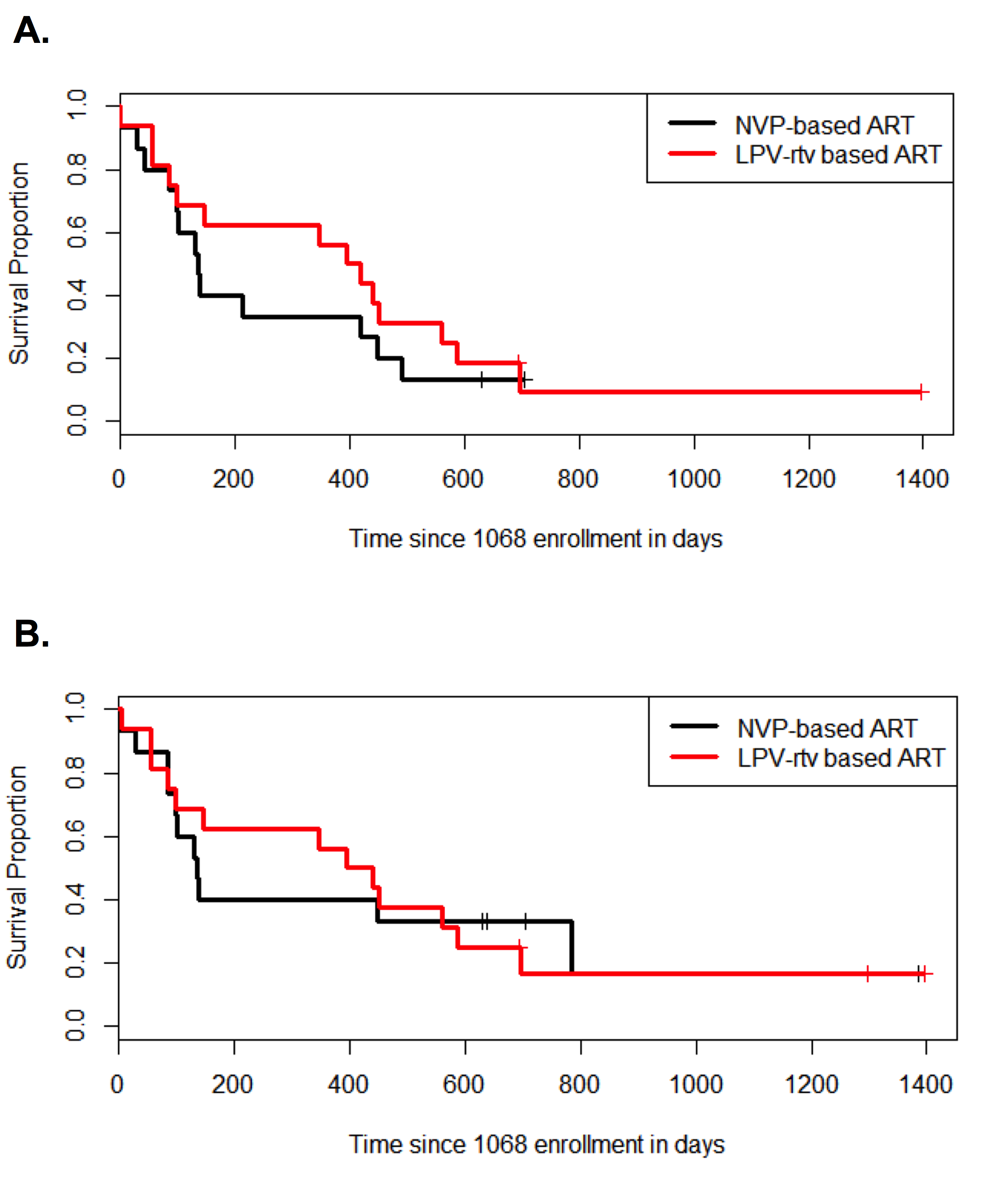

Supplement: S1 Fig — A and B: Kaplan Meier Curves depicting time to first (1st) positive blood smear (BS) and time to 1st confirmed clinical malaria (CCM) case. Overall, we observe a BS positive rate of 0.55 and 0.87 for the 1st year and the 1st two years of follow-up, respectively; for CCM rates are, 0.52 and 0.77 for the 1st year and the 1st two years, respectively. We observe 123 positive BS events, and 109 CCM events over the 97.89 observed person-years. In 1A, shown is the survival proportion and times to 1st positive BS. The median survival times are 408 and 135 days in the randomized to-LPV-rtv ART and randomized to-NVP ART groups, respectively. However, there was an 0.88 infection rate in the LPV-rtv ART group, and an 0.87 rate in the NVP-ART group over follow-up. In 1B, shown is the survival proportion and times to 1st CCM. The median time to 1st CCM, is 417 and 135 days in the randomized to-LPV-rtv ART and randomized to-NVP ART groups, respectively. However, there was an 0.81 CCM rate in the LPV-rtv group, and a 0.73 rate in the NVP-based group over follow-up. Note that for BS, the longest period while on NVP-ART prior to 1st positive BS is shorter compared with LPV-ART, and the figure incorporates subject censoring. (TIFF) [file pone.0165140.s001.tiff]
