# Supplementary material for: Malaria in HIV-Infected Children Receiving HIV Protease-Inhibitor- Compared with Non-Nucleoside Reverse Transcriptase Inhibitor-Based Antiretroviral Therapy, IMPAACT P1068s, Substudy to P1060
Source: PLoS One. 2016 Dec 9;11(12):e0165140. doi: 10.1371/journal.pone.0165140 (PMC5147802; doi:10.1371/journal.pone.0165140)
Supplement: S1 File — (DOC) [file pone.0165140.s002.doc]

**S1 File: Methods, Real Time PCR.** To identify *P. falciparum* parasites blood samples, real time, quantitative PCR was used as previously described, with previously published primers and with few modifications [16,17]. Parasite genomic DNA was isolated from Whatman 903 (Florham Park, NJ) dried blood spots using a QIAamp DNA mini (Qiagen) DNA extraction kit with the proteinase K step included for DNA extraction from dried blood spots. Three, 3 mm hole punches were made from each spot and used for DNA extraction. Cycling conditions as follows: 50º C for 20 min, 95º C for 15 min, 50 cycles of 94º C for 45 sec, 60º C for 45 sec. Previously published genus- and species-specific primers Taqman probe and pre-made kits were used [17]. An internal PCR control plasmid [16] was spiked into the samples prior to DNA isolation to control for PCR inhibition. Primers were designed to amplify this internal control (IPC Primer sequences: Forward: 5'-GTT AAG GGA GTG AAG ACG ATC AGA-3', Reverse: 5'-AAC CCA AAG ACT TTG ATT TCT CAT AA-3' IPC Probe sequence: 5'-CTC TCC GGA GAT TAG AACTCT TAG ATT GCT-3'). Failure to amplify this control flagged potential false negatives. In addition, a DNA standard curve was run with each experiment for quantification. Calculated PCR sensitivity was 0.045 parasites/μl. Negative samples were defined as less than 5 copies/L, based on 4-8 copies of Pf 18ssRNA per genome [16].
