# Supplementary material for: Malaria in HIV-Infected Children Receiving HIV Protease-Inhibitor- Compared with Non-Nucleoside Reverse Transcriptase Inhibitor-Based Antiretroviral Therapy, IMPAACT P1068s, Substudy to P1060
Source: PLoS One. 2016 Dec 9;11(12):e0165140. doi: 10.1371/journal.pone.0165140 (PMC5147802; doi:10.1371/journal.pone.0165140)
Supplement: S2 File — (DOC) [file pone.0165140.s003.doc]

**S2 File: Additional Statistical Analysis.**

**As Randomized Analysis (also refers to Tables S1A and S1B).**Thirty-two of 288 subjects from the P1060 parent study enrolled in P1068s, and 8 of these 31 children changed regimens before or after enrollment. For this reason we attempted to link observed treatment and observed outcomes. In addition, we provide herein as an-randomized analysis for the P1060 parent study, which found no significant associations.

Comparisons of time to first event of Positive BS and CCM were made with the use of a Cox proportional-hazards models; adjusted and unadjusted models were fit. For all adjusted versions of these models, CD4% at enrollment, sex, and age at enrollment (in months) were considered. Results are shown in Table S1A and are qualitatively similar to the as-treated analysis presented in the manuscript, but with higher p-values.

Similarly, as-randomized comparisons of the rate of Positive BS and CCM were made with the use of a negative binomial model; adjusted and unadjusted models were fit. For the adjusted versions of these models CD4% at enrollment, sex, age at enrollment (in months) were again considered. Results are shown in S1B. Like the as-randomized recurrent event analysis, these results are qualitatively similar to the as-treated analysis presented in the manuscript, but with higher p-values.

**Per-Protocol Analysis (also refers to Table S2).** Per-Protocol analysis of rates of BS and CCM were performed using a negative binomial model, offset for months on P1068s, with data censored at the point of a switch from the ART regimen from the as randomized regimen per P1060. We found some evidence to support an association between LPV-rtv ARV and fewer positive BS and CCM events using the negative binomial analysis when all data for a given subject were included prior to switching regimens. For BS the estimated RR for LPV-rtv ART was 0.54 in the fully adjusted model, p=0.08. For CCM, the estimated RR for LPV-rtv ARV was 0.56 for the fully adjusted model, p=0.09. This analysis removes 2 subjects from the group of 31, reducing our power to detect differences. The results of the adjusted and unadjusted versions of the censored models can be seen in Table S2.
