# Supplementary material for: Malaria in HIV-Infected Children Receiving HIV Protease-Inhibitor- Compared with Non-Nucleoside Reverse Transcriptase Inhibitor-Based Antiretroviral Therapy, IMPAACT P1068s, Substudy to P1060
Source: PLoS One. 2016 Dec 9;11(12):e0165140. doi: 10.1371/journal.pone.0165140 (PMC5147802; doi:10.1371/journal.pone.0165140)
Supplement: S1 Table — As-randomized in P1060 analyses (A. Cox Model and B. Negative Binomial). (DOC) [file pone.0165140.s004.doc]

**S1 Table: As-randomized in P1060 analyses A. Cox Model and B. Negative Binomial.**

1. **As Randomized Recurrent Events of Positive Blood Smears (BS) and Confirmed Clinical Malaria (CCM) (Cox Model).***

| **Positive BS** | | | | | | | **CCM** | | | | | |
| --- | --- | --- | --- | --- | --- | --- | --- | --- | --- | --- | --- | --- |
| **Unadjusted** | | | | **Adjusted** | | | **Unadjusted** | | | **Adjusted** | | |
|  | HR | 95% CI | p-value | HR | 95% CI | p-value | HR | 95% CI | p-value | HR | 95% CI | p-value |
| LPV-rtv ARV P1060 Randomized | 0.74 | (0.35,1.59) | 0.44 | 0.56 | (0.24,1.34) | 0.19 | 0.99 | (0.44,2.22) | 0.98 | 0.85 | (0.36,2.02) | 0.71 |
| CD4% at Enrollment |  |  |  | 1.02 | (0.98,1.05) | 0.42 |  |  |  | 1.02 | (0.98,1.06) | 0.26 |
| Enrollment Age (Months) |  |  |  | 0.96 | (0.93,1) | 0.04 |  |  |  | 0.97 | (0.94,1.01) | 0.13 |
| Sex (female) |  |  |  | 1.07 | (0.48,2.38) | 0.88 |  |  |  | 1.25 | (0.55,2.84) | 0.6 |

* Additional models were run but only unadjusted and fully adjusted results shown

**B. As Randomized Rates of Positive Positive Blood Smears (BS) and Confirmed Clinical Malaria (CCM) (Negative Binomial Model).***

| **Positive BS** | | | | | | | **CCM** | | | | | |
| --- | --- | --- | --- | --- | --- | --- | --- | --- | --- | --- | --- | --- |
| **Unadjusted** | | | | **Adjusted** | | | **Unadjusted** | | | **Adjusted** | | |
|  | RR | 95% CI | p-value | RR | 95% CI | p-value | RR | 95% CI | p-value | RR | 95% CI | p-value |
| LPV-rtv ARV P1060 Randomized | 0.85 | (0.44,1.64) | 0.63 | 0.61 | (0.33,1.14) | 0.12 | 0.94 | (0.46,1.92) | 0.86 | 0.69 | (0.35,1.34) | 0.27 |
| CD4% at Enrollment |  |  |  | 1.06 | (1.02,1.09) | <0.001 |  |  |  | 1.05 | (1.02,1.09) | <0.001 |
| Enrollment Age (Months) |  |  |  | 1.01 | (0.98,1.05) | 0.49 |  |  |  | 1.02 | (0.98,1.06) | 0.44 |
| Sex (female) |  |  |  | 1.3 | (0.73,2.32) | 0.38 |  |  |  | 1.39 | (0.73,2.64) | 0.31 |
| Enrollment Time between P1060 and P1068s |  |  |  | 0.93 | (0.88,0.99) | 0.03 |  |  |  | 0.93 | (0.87,1) | 0.04 |

* Additional models were run but only unadjusted and fully adjusted results shown
