# Supplementary material for: Malaria in HIV-Infected Children Receiving HIV Protease-Inhibitor- Compared with Non-Nucleoside Reverse Transcriptase Inhibitor-Based Antiretroviral Therapy, IMPAACT P1068s, Substudy to P1060
Source: PLoS One. 2016 Dec 9;11(12):e0165140. doi: 10.1371/journal.pone.0165140 (PMC5147802; doi:10.1371/journal.pone.0165140)
Supplement: S2 Table — (DOC) [file pone.0165140.s005.doc]

**S2 Table: Per Protocol Rates of BS and CCM (Negative Binomial Model, Data Censored at Switch from Regimen As Randomized).***

| **Positive BS**** | | | | | | | **CCM**** | | | | | |
| --- | --- | --- | --- | --- | --- | --- | --- | --- | --- | --- | --- | --- |
| **Unadjusted** | | | | **Adjusted** | | | **Unadjusted** | | | **Adjusted** | | |
|  | RR | 95% CI | p-value | RR | 95% CI | p-value | RR | 95% CI | p-value | RR | 95% CI | p-value |
| LPV-rtv ARV P1060 Randomized | 0.73 | (0.33,1.6) | 0.43 | 0.54 | (0.27,1.07) | 0.08 | 0.75 | (0.34,1.66) | 0.48 | 0.56 | (0.28,1.09) | 0.09 |
| CD4% at Enrollment |  |  |  | 1.06 | (1.03,1.1) | <0.001 |  |  |  | 1.06 | (1.02,1.1) | <0.001 |
| Enrollment Age (Months) |  |  |  | 1.01 | (0.97,1.05) | 0.67 |  |  |  | 1.01 | (0.97,1.05) | 0.67 |
| Sex (female) |  |  |  | 1.36 | (0.7,2.65) | 0.37 |  |  |  | 1.45 | (0.75,2.78) | 0.27 |
| Months on P1060 before P1068s |  |  |  | 0.92 | (0.86,0.99) | 0.03 |  |  |  | 0.92 | (0.86,0.99) | 0.03 |

* Additional models were run but only unadjusted and fully adjusted results shown

** Offset for months on P1068s
